# Programming and supervision of resistance training leads to positive effects on strength and body composition: results from two randomised trials of community fitness programmes

**DOI:** 10.1186/s12889-018-5289-9

**Published:** 2018-03-27

**Authors:** Steven Mann, Alfonso Jimenez, James Steele, Sarah Domone, Matthew Wade, Chris Beedie

**Affiliations:** 1 ukactive Research Institute, 4th and 5th Floor, 26-28 Bedford Row, London, WC1R 4HE UK; 2 Centre for Applied Biological and Exercise Sciences, Faculty of Health and Life Sciences, Coventry University, Coventry, UK; 3 School of Sport, Health, and Social Sciences, Southampton Solent University, Southampton, Hampshire SO14 0YN UK; 4 School of Sport, Health and Applied Science, St Mary’s University, Twickenham, TW1 4SX UK; 5 Department of Human & Life Sciences, Canterbury Christ Church University, Canterbury, Kent CTI IQU UK

**Keywords:** Resistance training, Body composition, Exercise treatment, Health status

## Abstract

**Background:**

Many sedentary adults have high body fat along with low fitness, strength, and lean body mass (LBM) which are associated with poor health independently of body mass. Physical activity can aid in prevention, management, and treatment of numerous chronic conditions. The potential efficacy of resistance training (RT) in modifying risk factors for cardiovascular and metabolic disease is clear. However, RT is under researched in public health. We report community-based studies of RT in sedentary (Study 1), and overweight and pre-diabetic (Study 2) populations.

**Methods:**

Study 1 - A semi randomised trial design (48-weeks): Participants choosing either a fitness centre approach, and randomised to structured-exercise (STRUC, *n* = 107), or free/unstructured gym use (FREE, *n* = 110), or not, and randomised to physical-activity-counselling (PAC, *n* = 71) or a measurement only comparator (CONT, *n* = 76). Study 2 - A randomised wait list controlled trial (12-weeks): Patients were randomly assigned to; traditional-supervised-exercise (STRUC, *n* = 30), physical-activity-counselling (PAC, *n* = 23), either combined (COMB, *n* = 39), or a wait-list comparator (CONT, *n* = 54). Outcomes for both were BF mass (kg), LBM (kg), BF percentage (%), and strength.

**Results:**

Study 1: One-way ANCOVA revealed significant between group effects for BF% and LBM, but not for BF mass or strength. Post hoc paired comparisons revealed significantly greater change in LBM for the STRUC group compared with the CONT group. Within group changes using 95%CIs revealed significant changes only in the STRUC group for both BF% (− 4.1 to − 0.9%) and LBM (0.1 to 4.5 kg), and in FREE (8.2 to 28.5 kg) and STRUC (5.9 to 26.0 kg) for strength.

Study 2: One-way ANCOVA did not reveal significant between group effects for strength, BF%, BF mass, or LBM. For strength, 95%CIs revealed significant within group changes for the STRUC (2.4 to 14.1 kg) and COMB (3.7 to 15.0 kg) groups.

**Conclusion:**

Strength increased in both studies across all RT treatments compared to controls, yet significant improvements in both strength and body-composition occurred only in programmed and/or supervised RT. As general increases in physical activity have limited impact upon body-composition, public health practitioners should structure interventions to include progressive RT.

**Trial registration:**

Study 1: ISRCTN13024854, retrospectively registered 20/02/2018. Study 2: ISRCTN13509468, retrospectively registered 20/02/2018).

## Background

Reducing population-level physical inactivity has been identified as a key intervention in public health [1]. In this context, research and public health messaging tends to centre on habitual low-moderate intensity of effort aerobic activity such as active transport, walking and cycling, or purposeful low-moderate intensity aerobic activity such as recreational sport, jogging or swimming [2, 3]. Such activity has been described as ‘the gold standard for health professionals when prescribing exercise programmes’ [4]. This is likely the case because such aerobic activities are, hypothetically at least, effective, safe, widely accessible, and associated with few legitimate barriers to participation for the majority of individuals. Indeed, they are the primary component of guidelines for physical activity from the World Health Organisation (WHO) [5].

The health problems associated with excess body fat (BF) are well documented [6]. Over and above high BF however, many sedentary adults also have low fitness, strength, and lean body mass (LBM) all of which have been shown to be associated with poor health and longevity independently of body mass [6–8]. Whilst health risks associated with the former are widely accepted, those associated with the latter, such as increased likelihood of Type-2 Diabetes, are less well recognized. The independent and combined role of muscle function and muscle mass in disease prevention and management is however increasingly evident. For example, recent data indicate that high muscle strength is associated with lower cancer mortality risk [9] and lower risk of arrhythmia [10], whilst low LBM is associated with hyperglycemia [11] and higher mortality risk in obese men [12]. Furthermore, and irrespective of disease risk, across the lifespan but especially in old age, appropriate LBM helps maintains mobility, balance, and injury resilience, and thereby maintains independence and quality of life [13, 14].

In individuals with normal/healthy LBM, healthy body composition can be maintained via the modulation of fat mass through aerobic exercise and/or dietary means (although diet-induced fat loss alone can reduce LBM as well as fat mass, an effect not necessarily observed when exercise alone is used to induce fat loss [15, 16]). However, many adults have below optimal LBM, and this tends to become more pronounced with increasing age, whilst fat mass tends to accrue at the same time. Further, there is a loss of muscle quality affecting components of muscle function such as strength. Such individuals require interventions to increase LBM and muscle function [17].

Aerobic exercise at the intensity of effort often promoted in the public health context is expected to produce positive effects on BF (and indeed cardiorespiratory health and muscle endurance). However, few positive effects are expected or observed on either muscle strength and/or muscle mass [18]. In fact, in the public health context, muscle function and muscle morphology are often considered of secondary importance to broader cardiovascular and metabolic function. Muscle function and mass have historically been viewed more as components of ‘athletic fitness’ than of public health [2], a scenario referred to as the ‘underappreciated role of muscle in health and disease’ [19]. Yet, there is considerable evidence accumulating that both greater strength and muscle mass are associated health and longevity [3]. Recommendations to engage in ‘muscle strengthening activities’ such as resistance training (RT) are currently included in the WHO physical activity guidelines [5]. However, in comparison to the aerobic physical activity recommendations, these lack emphasis. As a result, numerous authors have argued that higher effort interventions such as RT should have a more prominent place within public health approaches towards physical activity and exercise [2, 3, 20, 21]. It is unfortunate however that RT is underused and under-researched in public health.

The potential efficacy of RT in modifying risk factors for cardiovascular and metabolic disease has been demonstrated [4, 22]. Data from two recent large studies conducted in community settings indicate that of several modes of physical activity, RT was associated with the lowest increases in waist circumference over a 12-year period [23] and that adults with excess BF benefitted particularly from RT [24]. Further, the Resist Diabetes trial demonstrated that low volume yet high effort RT was an effective and maintainable approach for increasing strength and reducing prevalence of pre-diabetes [25]. Recent systematic reviews support this indicating that RT conducted at sufficiently high intensities of effort (either through increased loads, repetitions, or sets) was associated with improved insulin sensitivity [26] and concluded that RT presents a viable alternative and adjunct to aerobic exercise in the management of hypercholesterolemia [27].

However, much of what we know about the effects of RT is derived from research in sports science and sports medicine, which has identified, for example, the effects of manipulation of variables (load, volume, effort etc.) within RT interventions in various healthy and/or athletic populations [28, 29]. In public health however, many of the nuances of sports-related RT may be less relevant. In public health, evidence that an intervention is broadly effective for a broad range of individuals, and that it might be robust in the face of variations in delivery, environment and demographic are critical. In fact, in public health it is the commonalities across intervention effects, not the nuanced differences between them, that are important. What we need to know in the public health context is what characteristics of a RT programme make it an effective health intervention in normal, at risk, and diseased populations. In the process of identifying the answer to such questions, many aspects of sports-related RT may of course become significant.

Recent government reports, for example the All Party Commission on Physical Activity [1], ‘Tackling physical inactivity - a coordinated approach’, public health reports, for example ‘Identifying what works for local physical inactivity interventions’ [30], and published academic papers [31], have identified a lack of data attesting to the effectiveness of real world physical activity interventions. Beedie et al. [31] argued that whilst the evidence for the laboratory efficacy of exercise is strong, the evidence for its effectiveness in real world public health contexts is weak. If this statement is true in the case of exercise generally, we argue that it is especially true in the case of RT [3].

As such, the aim of the present paper is to report two community-based RT interventions, delivered to a previously sedentary population (Study 1), and as part of a GP Exercise Referral programme to overweight and pre-diabetic patients (Study 2). We report these two studies together here for two reasons; first, the two were linked in that promising data derived from an inactive yet healthy population in Study 1 enabled us to test a similar model on a less healthy population in Study 2. Second, we believe that the commonalities across findings and complementary conclusions are worthy of joint dissemination. Data reported were collected as part of larger projects examining community-based exercise interventions in public health.

## Study 1: Effects on strength and body composition of prescribed and structured versus free resistance training

### Study 1 method

#### PICO and trial design

**Fig. 1:**
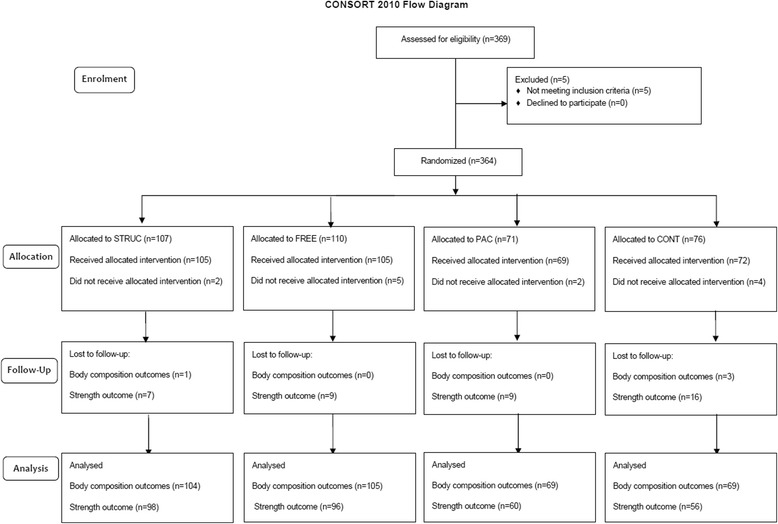
CONSORT flow diagram for Study 1

The population (P) was sedentary adults. The interventions (I) included two fitness centre interventions and a physical activity counselling intervention both described below, and the comparator (C) was a measurement only control group. Outcomes (O) included body composition and strength. A semi-randomised trial design was utilised. Participants were initially offered one of two pathways. Those choosing the fitness centre pathway were randomised to one of two interventions; a structured exercise programme (STRUC), or free/unstructured exercise (FREE). Those choosing a non-fitness centre pathway were randomised to either physical activity counselling (PAC), or to a measurement only control condition (CONT) including two health checks. Interventions were delivered over 48 weeks with measures at 0 (baseline) and 48 weeks. Ethical approval was granted from the institution of the lead author (University of Greenwich, UK, UREC/11/12.5.6.11). All participants gave consent for publication. Fig. 1 shows the CONSORT flow diagram for study 1. The trial was retrospectively registered on the ISRCTN Registry (ISRCTN13024854).

#### Recruitment

Operators of community health centres in the UK were invited to participate in the study. Two exercise professionals from each of 27 participating facilities (*n* = 54) were trained in a 2-day bespoke course delivered by the first author. Each centre was tasked with recruiting sedentary participants to the project. In order to maintain the external validity of the study, centres were informed that no recruitment incentives were to be offered [32].

#### Participants

Inclusion criteria for participants were that they were sedentary, defined as currently not meeting the physical activity recommendations of the UK Chief Medical Officer, and were taking no medication that might impact cardiovascular risk. Three hundred and sixty-nine participants (age 43 ± 5 years) were recruited. Participants received a detailed explanation of the study and provided written informed consent.

#### Interventions & comparator

STRUC had access to all fitness centre facilities and received an individualised and structured RT programme (Table 1). This programme was based on guidelines published by the American College of Sports Medicine (ACSM) [33, 34]. RT loads were based upon calculations of one-repetition maximum (1RM) derived from baseline data (see below). As the studies were conducted in ecologically valid community settings there was some flexibility in the exercises utilised based on participant preferences and any orthopaedic issues/injuries. However, all participants at a minimum followed a full body routine consisting of an upper body multi-joint push (e.g. chest press, overhead press, or dip), upper body multi-joint pull (e.g. pulldown, or seated row), and lower body multi-joint push (e.g. leg press). Exercise professionals met STRUC participants once a month to discuss their progress.

**Table 1:** Periodized resistance training programme for structured (STRUC) participants

Mesocycle 1	Week 1–3 1 × 8–10 reps (70% 1RM)	Week 3–5 2 × 15 reps (40% 1RM)	Weeks 6–8 3 × 12 reps (50% 1RM)	Week 9–12 4 × 12 reps (50% 1RM)
Mesocycle 2	Week 13–16 4 × 10 reps (60% 1RM)		Week 17–24 3 × 15 reps (40% 1RM)	
Mesocycle 3	Week 25–27 4 × 10 reps (60% 1RM)		Week 28–36 3 × 12 reps (50% 1RM)	
Mesocycle 4	Week 37–39 3 × 6 reps (80% 1RM)		Week 40–48 4 × 10 reps (60% 1RM)	

FREE participants had access to all fitness centre facilities but received no structured programme. Exercise professionals met with FREE participants once each month to discuss progress.

PAC participants met exercise professionals once each month for counselling sessions structured around the model proposed by Haase et al. [35] and delivered within the fitness centre location. PAC participants did not however have access to any fitness centre exercise facilities.

CONT participants acted as the comparator group, did not receive an intervention, and did not have access to any fitness centre exercise facilities. Whilst CONT did not receive an exercise intervention, they did receive two free health screens (pre and post measurement) over the duration of the study. Exercise professionals were instructed to have no contact with CONT participants other than to arrange data collection at 0 and 48 weeks.

#### Outcomes

Pre- and post- intervention measures of body composition including BF mass (kg), LBM (kg) and BF percentage (%) were performed using bioelectrical-impedance (Bodystat 1500, Bodystat, Isle of Man, UK). Guidelines from the National Institute of Health Research Southampton Biomedical Research Centre were followed for body compositions assessment (http://www.uhs.nhs.uk/Media/Southampton-Clinical-Research/Procedures/BRCProcedures/Procedure-for-bioimpedance-with-Bodystat-1500.pdf). Predicted 1RM for chest press, pull down, and leg press were obtained by gauging the maximal weight that could be lifted successfully for between 5 and 15 repetitions, and inputting these data into the Brzycki equation (i.e. weight/(1.0278-(0.0278 x No. Repetitions)) [36]. These results were collapsed into a single strength measure (the mean of the predicted 1RM for each exercise). No direct measures of physical activity were employed.

#### Data analysis

The independent variable considered in the analysis was ‘group’ i.e. FREE, PAC, STRUC, or CONT) and the dependent variables were the absolute changes (post- minus pre-test values) for changes in strength and body composition. Between group comparisons were made using one-way analysis of covariance (ANCOVA) using the pre-test results as a covariate in the model. Paired comparisons for significant between group effects were examined using post hoc Bonferroni tests. Within group changes were examined using 95% confidence intervals (CIs) for marginal means from ANCOVA group model with a Bonferonni adjustment, and where significant within participants effects were detected, effect  sizes  (ES; *d* = μ_change_*/σ*_change;_ marginal = < 0.20, small = 0.20–0.49, moderate = 0.50–0.79, and large = ≥ 0.80) were calculated. Analysis was conducted using JASP (version 0.8.1.2; University of Amsterdam, The Netherlands) with α for statistical significance set at 0.05.

### Study 1 results

All pre- and post-intervention means±SD, marginal means for changes, and 95%CIs for changes are reported in Table 2.

**Table 2:** Pre- and post-intervention means±SD, marginal means for changes, and 95%CIs for strength and body composition in Study 1

Variable	Pre- (Mean ± SD)	Post- (Mean ± SD)	Change (Marginal Means)	95% Confidence Interval for Change
Strength (kg)				
CONT	67.0 ± 22.4	72.2 ± 37.0	4.4	−8.9 to 17.7
FREE	71.0 ± 27.4	91.4 ± 56.4	18.4	8.2 to 28.5
PAC	71.4 ± 28.0	79.3 ± 36.2	7.2	−5.9 to 20.3
STRUC	66.4 ± 24.4	87.0 ± 45.5	16.0	5.9 to 26.0
BF%				
CONT	34.2 ± 11.5	34.3 ± 11.7	0.1	−1.9 to 2.1
FREE	34.7 ± 14.8	34.2 ± 14.9	−0.5	−2.1 to 1.1
PAC	34.9 ± 13.0	34.2 ± 13.4	−0.8	−2.7 to 1.3
STRUC	35.7 ± 16.0	32.9 ± 15.1	−2.5	−4.1 to −0.9
BF Mass (kg)				
CONT	24.8 ± 13.4	23.1 ± 9.3	−1.5	−3.1 to 0.2
FREE	24.4 ± 11.9	23.7 ± 11.3	−0.8	−2.1 to 0.6
PAC	25.1 ± 9.8	24.4 ± 9.0	−0.6	−2.2 to 1.1
STRUC	24.7 ± 10.8	23.1 ± 10.3	−1.3	−2.6 to 0.0
LBM (kg)				
CONT	49.0 ± 18.6	47.1 ± 18.6	−1.8	−4.5 to 0.9
FREE	48.9 ± 18.2	48.5 ± 18.2	−0.4	−2.6 to 1.8
PAC	50.3 ± 18.4	51.4 ± 18.9	1.2	−1.5 to 3.9
STRUC	48.4 ± 19.4	50.7 ± 18.1	2.3	0.1 to 4.5

*CONT* control, *FREE* free/unstructured exercise, *PAC* physical activity counselling, *STRUC* structured exercise programme

For change in strength, one-way ANCOVA did not reveal significant between group effects (*F*_(3,303)_ = 2.064, *p* = 0.105). However, 95%CIs revealed that significant within group changes occurred only for the FREE (8.2 to 28.5 kg; *p* < 0.001) and STRUC (5.9 to 26.0 kg; *p* < 0.001) groups. ESs for change in strength were marginal for both CONT (*d* = 0.11) and PAC (*d* = 0.18), and were small for both FREE (*d* = 0.46) and STRUC (*d* = 0.40).

For changes in body composition, one-way ANCOVA revealed significant between group effects for BF% (*F*_(3,342)_ = 2.739, *p* = 0.043) and LBM (*F*_(3,342)_ = 3.511, *p* = 0.016), but not for BF mass (*F*_(3,342)_ = 0.517, *p* 0.671). Post hoc paired comparisons for BF% were not significant for any comparisons but revealed significantly greater change in LBM for the STRUC group compared with the CONT group (*p* = 0.019). Within group changes in body composition using 95%CIs revealed significant changes only in the STRUC group for both BF% (− 4.1 to − 0.9%; *p* < 0.001) and LBM (0.1 to 4.5%; *p* = 0.032). ESs for change in BF% were marginal for CONT (*d* = 0.02), FREE (*d* = − 0.07), and PAC (*d* = − 0.11), and small for STRUC (*d* = − 0.38). ESs for change in LBM were marginal for CONT (*d* = − 0.20), FREE (*d* = − 0.05), and PAC (*d* = 0.14), and small for STRUC (*d* = 0.26).

## Study 2: Effects on strength and body composition of structured and supervised resistance training, physical activity counselling and the two combined

### Study 2 method

#### PICO and trial design

**Fig. 2:**
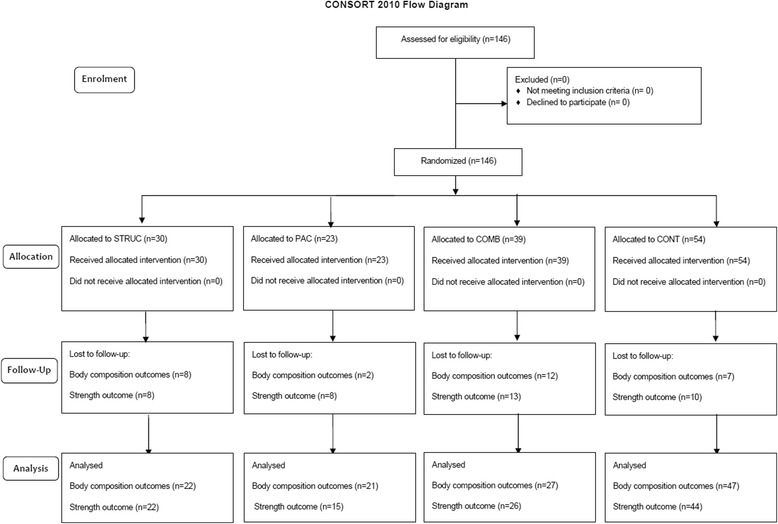
CONSORT flow diagram for Study 2

The population (P) was sedentary overweight or obese adults with, or at increased risk of, Type 2 Diabetes. The interventions (I) included three interventions groups described in detail below; a general practitioner (GP) exercise referral scheme of structured exercise (STRUC), physical activity counselling (PAC), or a combination of both (COMB). The comparator (C) was a wait-list control group awaiting entry into the GP exercise referral scheme. Outcomes (O) included body composition and strength. A randomised wait-list controlled trial was utilised. All interventions were delivered over a period of 12 weeks. Participants were randomly assigned to one of four groups including the three intervention groups and one wait-list control group. Ethical approval was granted from the local NHS research ethics committee (IRAS project ID 172321, REC reference: 15/LO/0540). Fig. 2 shows the CONSORT flow diagram for study 2. The trial was retrospectively registered on the ISRCTN Registry (ISRCTN13509468).

#### Participants

Inclusion criteria for participants were that they were overweight and/or obese (BMI 25–35), and/or at increased risk of Type 2 Diabetes as determined by their General Practitioner (GP), yet not currently taking any prescribed medication for cardiovascular or metabolic conditions. Following local NHS research ethics committee approval, letters were sent by the research team inviting all GPs in the region to identify and contact potential participants. One hundred and forty-six participants (age 49 ± 14 years) who were residents of South-East London, UK were recruited. All participants signed informed consent documents and all gave consent for publication. Those taking prescribed medication for cardiovascular or metabolic conditions were excluded from the study, but were referred into the non-research arm of the treatment.

#### Interventions & comparator

STRUC received one session per week of a structured and supervised GP exercise referral intervention. This intervention was already delivered as part of the care pathway of the local health trust and participants were restricted to these sessions. This programme (Table 1) was based on guidelines published by the American College of Sports Medicine (ACSM) [33, 34]. RT loads were based upon calculations of one-repetition maximum (1RM) derived from baseline data (see below). As the studies were conducted in ecologically valid community settings there was some flexibility in the exercises utilised based on participant preferences and any orthopaedic issues/injuries. However, all participants at a minimum followed a full body routine consisting of an upper body multi-joint push (e.g. chest press, overhead press, or dip), upper body multi-joint pull (e.g. pulldown, or seated row), and lower body multi-joint push (e.g. leg press). Exercise professionals met STRUC participants once a month to discuss their progress.

PAC received one session per week of physical activity counselling. The sessions were structured around the model proposed by Haase et al. [35], and no access to fitness facilities.

COMB received a combination of physical activity counselling (sessions in weeks 1, 3, 5, 7, 9 & 11) and a structured and supervised GP exercise referral intervention (sessions in weeks 2, 4, 6, 8, 10 & 12).

The CONT group was formed from a wait-list control facilitated by a legitimate 12-week waiting list for entry into the GP exercise referral intervention. CONT participants received the intervention after this period, though only their waiting list period data was included for analysis.

#### Outcomes

Pre- and post-intervention measures for body composition including, BF mass (kg), lean mass (kg) and BF percentage (%), were performed using bio-impedance (Bodystat 1500, Bodystat, Isle of Man, UK). Guidelines from the National Institute of Health Research Southampton Biomedical Research Centre were followed for body compositions assessment (http://www.uhs.nhs.uk/Media/Southampton-Clinical-Research/Procedures/BRCProcedures/Procedure-for-bioimpedance-with-Bodystat-1500.pdf). Predicted 1RM for chest press, pull down and leg press were obtained by gauging the maximal weight that could be lifted successfully for between 5 and 15 repetitions, and inputting these data into the Brzycki equation (i.e. weight/(1.0278-(0.0278 x No. Repetitions)) [36]. These results were collapsed into a single strength measure (the mean of the predicted 1RM for each exercise). No direct measures of physical activity were employed.

All interventions and measures were conducted by the exercise staff of the three sites, all of whom were qualified and experienced exercise professionals. All staff were trained to deliver PAC and conduct all measures by the research team. The research team however had no direct contact with participants at any stage of the study.

#### Data analysis

The independent variable considered in the analysis was ‘group’ i.e. STRUC, PAC, COMB, or CONT) and the dependent variables were the absolute changes (post- minus pre-test values) for changes in strength and body composition. Between group comparisons were made using one-way analysis of covariance (ANCOVA) using the pre-test results as a covariate in the model. Paired comparisons for significant between group effects were examined using post hoc Bonferroni tests. Within group changes were examined using 95%CIs for marginal means from ANCOVA group model with a Bonferonni adjustment, and where significant within participant effects were detected, effect size (ES; *d* = μ_change_*/σ*_change;_ marginal = < 0.20, small = 0.20–0.49, moderate = 0.50–0.79, and large = ≥ 0.80) was calculated. Analysis was conducted using JASP (version 0.8.1.2; University of Amsterdam, The Netherlands) with α for statistical significance set at 0.05.

### Study 2 results

All pre- and post-intervention means±SD, marginal means for changes, and 95%CIs for changes are reported in Table 3.

**Table 3:** Pre- and post-intervention means±SD, marginal means for changes, and 95%CIs for strength and body composition in Study 2

Variable	Pre- (Mean ± SD)	Post- (Mean ± SD)	Change (Marginal Means)	95% Confidence Interval for Change
Strength (kg)				
STRUC	28.8 ± 13.6	37.6 ± 12.9	8.2	2.4 to 14.1
COMB	41.7 ± 18.8	48.3 ± 19.0	9.4	3.7 to 15.0
PAC	24.7 ± 10.2	31.8 ± 9.8	5.2	−1.9 to 12.4
CONT	29.3 ± 14.4	32.8 ± 15.3	2.8	−1.3 to 7.0
BF%				
STRUC	35.5 ± 12.5	35.7 ± 14.0	−1.1	−3.1 to 0.9
COMB	38.7 ± 12.4	36.5 ± 8.5	−0.3	−2.1 to 1.6
PAC	38.9 ± 9.9	38.6 ± 8.9	−0.1	−2.2 to 2.0
CONT	37.2 ± 9.3	35.9 ± 9.3	−0.3	−1.7 to 1.2
BF Mass (kg)				
STRUC	32.3 ± 19.3	33.7 ± 21.5	−1.8	−4.3 to 0.7
COMB	37.5 ± 16.0	36.6 ± 15.0	−0.1	−2.5 to 2.2
PAC	35.2 ± 14.0	32.4 ± 10.7	−2.0	−1.6 to 0.7
CONT	34.0 ± 13.5	31.0 ± 11.8	−0.7	−2.5 to 1.2
LBM (kg)				
STRUC	56.1 ± 14.5	56.0 ± 12.0	−1.4	−5.7 to 3.0
COMB	58.9 ± 13.0	62.9 ± 14.4	2.6	−1.3 to 6.5
PAC	48.5 ± 12.6	49.8 ± 9.5	1.3	−3.3 to 5.9
CONT	55.5 ± 13.2	55.7 ± 13.9	0.5	−2.3 to 3.4

*STRUC* traditional-supervised-exercise, *PAC* physical-activity-counselling, *COMB* combination of traditional-supervised-exercise and physical-activity-counselling, *CONT* wait-list control

For change in strength, one-way ANCOVA did not reveal significant between group effects (*F*_(3,3102)_ = 2.319, *p* = 0.080). However, 95%CIs revealed that significant within group changes occurred only for the STRUC (2.4 to 14.1 kg; *p* = 0.002) and COMB (3.7 to 15.0 kg; *p* < 0.001) groups. ESs for change in strength were small for both CONT (*d* = 0.26) and PAC (*d* = 0.48), and were moderate for both STRUC (*d* = 0.76) and COMB (*d* = 0.76).

For changes in body composition, one-way ANCOVA did not reveal significant between group effects for BF% (*F*_(3,112)_ = 0.346, *p* = 0.792), BF mass (*F*_(3,111)_ = 0.876, *p =* 0.456), or LBM (*F*_(3,111)_ = 1.056, *p* = 0.371). Within group changes in body composition using 95%CIs revealed no significant changes.

## Discussion

RT interventions are under-utilised and under-researched in public health. Above we reported two studies conducted in community settings, both of which used existing service delivery infrastructure to examine the effects of RT in sedentary (Study 1) and at risk (Study 2) participants. Both studies indicate that when RT is included in delivery in a range of intervention modes, significant increases in strength occur. Consistent with recent reviews [2–4] this indicates that several forms of RT, ranging from free and unstructured to structured, periodized and supervised, have value in enhancing the functional capacity of sedentary and at-risk adults. This will in turn reduce risk of disease [9, 10] and in older individuals may maintain independence and reduce risk of injury [13, 14]. These effects would be expected independent of any observed changes in muscle mass, as in study 2 we found changes in strength but not body composition. Further, despite the lack of emphasis in current physical activity guidelines regarding participation in RT [5], recent evidence suggests that RT independent of aerobic exercise has the greatest impact on risk reduction for metabolic syndrome [37].

Significant strength changes were found within the arms of each study that included a RT component (e.g. FREE, STRUC in both Study 1 and 2, and COMB in Study 2) and the magnitude of these changes were very similar between the arms within each study (study 1; FREE, *d* = 0.46 vs. STRUC, *d* = 0.40; and study 2; STRUC, *d* = 0.76 vs. COMB, *d* = 0.76). Absolute changes were greater for study 1 (Table 2), though the greater ESs in study 2 might be expected due to the patient population examined, despite the far shorter duration of the intervention period compared with study 1 (12 weeks vs 48 weeks). Further, in study 2, participants were also directly supervised and it has been shown that direct supervision during RT may impact upon outcomes [38–43]. However, within study 1, both the FREE and STRUC groups had similar increases in strength. These findings suggest that, independently of structured programing, participation in unsupervised RT is likely to result in similar strength gains in previously untrained and inactive participants.

Despite strength gains being similar, in study 1 only the STRUC group had significant changes in body composition including decreases in BF% and increases in LBM. Multiple mechanisms, ranging from current energy balance and nutrient intake to previous training history and heredity, might underlie changes in body composition resulting from RT. Our data are not sufficient to identify which of these individually or in combination might explain observed effects. Interestingly, though strength changes may be less influenced, body composition appears to be more greatly influenced by supervision during RT [43]. Furthermore, more pronounced changes observed in some groups may simply be dose-dependent. In study 1 for example, STRUC participants were provided with a programme specifying RT over 48 weeks whilst those in FREE were not. FREE participants who lacked either the necessary motivation or aptitude might have completed little or no RT during the intervention period. The specific programming offered may have overcome the lack of direct supervision and thus resulted in the greater body composition changes (greater BF% decrease and LBM increase) observed. Likewise, in study 2 STRUC participants experienced twice as many supervised exercise sessions as COMB and experienced greater BF% decrease. Again this may be due to between-group differences in volumes of RT completed. Such a hypothesis is consistent with a recent meta-analysis that identified significant dose-response effects in RT performed by healthy older adults [44].

Having said this, we must of course return to the issue of public health messaging alluded to above; the question of whether effects are the result of tailoring, programming, and supervision, or are simply dose dependant, is perhaps moot in the sense that for many sedentary or at-risk individuals the former would almost certainly lead to the latter. That is, personal contact with, or supervision by, an exercise or health professional or their proxy (e.g., online resources) is likely a factor in subsequent exercise behaviour. Further, participation in structured RT may subsequently influence participation in other health promoting behaviours [45, 46]. In study 1, STRUC and FREE were randomly assigned, as were STRUC and COMB in study 2. We therefore have no reason to suspect any systematic differences in aptitude or motivation for RT between groups, and whether dose-dependent or not, structured and supervised RT was more effective in both studies. This is a useful finding, one that perhaps highlights that in health – as is often the case with sports-specific applications – RT might be more effective when structured and/or supervised by an exercise professional. It should however be noted that we did not assess the fidelity of the interventions employed and as such, though it seems that broad conclusions can be drawn with respect to the nature of the interventions (i.e. that supervised/structured RT may be more effective), it is difficult to comment on the specific nature of the interventions and the interaction that may have had with our results. For example, due to the nature of some of the groups (e.g. FREE) it would have proved difficult to track in any meaningfully way that could be compared with other groups (e.g. STRUC) on participants use of the fitness facilities in terms of frequency, duration, or nature. As such, this is a noted limitation of the present studies.

## Conclusion

Although increases in strength were observed across all RT treatments compared to controls, significant improvements in both strength and body-composition were observed only in programmed and/or supervised RT. Data suggests general increases in PA have limited impact upon body-composition in comparison with interventions including RT. Whilst the data presented are promising, future research will need to further examine potential dose-response relationships in community-based RT, and examine the effects of RT on a broader range of dependent variables. Furthermore, it should seek to examine both programme-based and individual difference factors likely to explain the relatively variable response to RT in such settings. Public health practitioners should structure PA interventions to include progressive RT.

## Abbreviations

ANCOVA: Analysis of Covariance
BF: Body Fat
COMB: Combination (of PAC and STRUC)
CONT: Control
FREE: Free/Unstructured Exercise
GPER: GP Exercise Referral
LBM: Lean Body Mass
PAC: Physical Activity Counselling
RT: Resistance training
STRUC: Structured Exercise Programme
